# Circulating free DNA as a predictive biomarker for response to nivolumab and platinum-based chemotherapy in metastatic esophageal adenocarcinoma: a prospective pilot study

**DOI:** 10.3389/fonc.2025.1678068

**Published:** 2025-11-03

**Authors:** Qing Sheng Du, Bo Fan

**Affiliations:** ^1^ Department of Thoracic Surgery, Shandong Provincial Third Hospital, Jinan, Shandong, China; ^2^ Department of Thoracic Surgery, The First Affiliated Hospital of Shandong First Medical University (Shandong Qianfoshan Hospital), Jinan, Shandong, China

**Keywords:** circulating free DNA, esophageal adenocarcinoma, immune checkpoint inhibitors, tumor mutational burden, treatment response

## Abstract

**Background:**

Reliable biomarkers are urgently needed to predict response to immune checkpoint inhibitors in metastatic esophageal adenocarcinoma (mEAC). This study evaluated early circulating free DNA (cfDNA) dynamics as a predictor of treatment response and survival in patients receiving platinum-based chemotherapy plus Nivolumab.

**Methods:**

In this prospective pilot study, 95 patients with mEAC were treated with Nivolumab and platinum-based chemotherapy. Plasma cfDNA levels were measured at baseline, Day 15, and Day 30 using digital droplet PCR. The primary outcome was objective treatment response; secondary outcomes included progression-free survival (PFS) and overall survival (OS). Tumor mutational burden (TMB), PD-L1 expression, liver metastasis, and ECOG status were also assessed.

**Results:**

Patients with a cfDNA Day 30/Baseline ratio <0.4 had significantly improved median PFS (11 vs. 4 months) and OS (14 vs. 7 months) compared to those with ratios >0.8 (p for trend <0.001). Early decline in cfDNA correlated with treatment response. High TMB (≥10 mut/Mb) was independently associated with increased response (adjusted OR: 2.5, 95% CI: 1.2–5.2, p=0.015). ECOG >1 was inversely associated with response (adjusted OR: 0.35, p=0.01). PD-L1 expression and liver metastasis were not significantly predictive.

**Conclusion:**

Early cfDNA kinetics—particularly a Day 30/Baseline ratio <0.4—strongly predicted response and survival in mEAC patients receiving chemoimmunotherapy. cfDNA monitoring offers a promising non-invasive tool for early treatment stratification and response assessment in this population.

## Introduction

Esophageal cancer remains one of the most aggressive and deadly malignancies worldwide, ranking sixth in cancer-related mortality (1). Among its subtypes, esophageal adenocarcinoma (EAC) has seen a rising incidence in Western countries, often presenting at an advanced stage with poor prognosis (2). Despite recent advances in systemic therapy, including the incorporation of immune checkpoint inhibitors, the overall survival for patients with metastatic EAC remains dismal, with a 5-year survival rate below 20% (3–5).

The standard first-line treatment for metastatic EAC has traditionally relied on platinum-based chemotherapy (6). However, the emergence of immunotherapy, particularly programmed death-1 (PD-1) inhibitors such as Nivolumab, has transformed the therapeutic landscape (3). Recent phase III trials, such as CheckMate 649 and ATTRACTION-4, have demonstrated survival benefits with PD-1 blockade in gastroesophageal cancers, leading to its regulatory approval in combination with chemotherapy (7, 8). Nevertheless, only a subset of patients derive durable benefit from immunotherapy, underscoring the urgent need for reliable predictive biomarkers to guide personalized treatment selection.

Several biomarkers have been explored to predict response to immune checkpoint inhibitors, including PD-L1 expression, tumor mutational burden (TMB), and microsatellite instability (MSI) (9). While these markers have shown promise, their predictive accuracy remains limited and inconsistent in esophageal cancer (10, 11). Moreover, they often require invasive tissue sampling, are static in nature, and may not capture real-time tumor dynamics.

In this context, circulating free DNA (cfDNA) has emerged as a promising non-invasive biomarker that can reflect tumor burden, monitor treatment response, and detect minimal residual disease (12). cfDNA consists of fragmented tumor-derived DNA shed into the bloodstream, and its levels can be dynamically quantified at multiple time points during therapy (13). Prior studies in colorectal, lung, and breast cancers have demonstrated that early reductions in cfDNA levels during treatment are associated with improved clinical outcomes, often preceding radiographic changes (14, 15). However, data on cfDNA kinetics in metastatic EAC, particularly in the setting of chemoimmunotherapy, remain scarce.

Recent studies highlight the potential of ctDNA as a prognostic and predictive biomarker in esophageal and gastroesophageal cancers, particularly for chemoimmunotherapy and perioperative settings (16–19). However, controversies persist regarding optimal detection methods, assay standardization, and threshold definitions for clinical utility in advanced disease.

This prospective pilot study aimed to assess the clinical utility of early cfDNA dynamics—specifically the change in cfDNA levels at days 15 and 30 from baseline—as predictive biomarkers of treatment response in patients with metastatic esophageal adenocarcinoma treated with Nivolumab and platinum-based chemotherapy. In addition, we examined the role of PD-L1 expression, TMB, liver metastasis, and ECOG performance status as potential predictive factors. We hypothesized that early decline in cfDNA, particularly by day 30, would correlate with objective treatment response and survival, and that cfDNA could serve as a practical tool for early response stratification in this patient population.

## Methods

### Study design and patient population

This was a prospective pilot study conducted at The First Affiliated Hospital of Shandong First Medical University, designed to evaluate the predictive utility of cfDNA dynamics in patients with mEAC undergoing first-line treatment with platinum-based chemotherapy plus Nivolumab.

Eligible participants were adults (≥18 years) with histologically confirmed mEAC, measurable disease per RECIST 1.1, ECOG performance status ≤2, and adequate organ function. Patients were required to have available archival tumor tissue for biomarker analysis and detectable cfDNA mutations identifiable at baseline. Patients with active autoimmune disorders, prior systemic therapy for metastatic disease, or untreated CNS metastases were excluded. Additional exclusion criteria included active infections, uncontrolled cardiovascular conditions, or history of other malignancies within the past 5 years (except adequately treated basal cell carcinoma or cervical carcinoma *in situ*).

This study has been approved by the Medical Ethics Committee of the First Affiliated Hospital of Shandong First Medical University (Shandong Qianfoshan Hospital) (Ethics Approval ID: 2025- Lun-Shen- Zi (S978)). Written informed consent was obtained from all participants prior to enrollment. All study procedures adhered to the ethical principles of the Declaration of Helsinki.

### Treatment protocol

All patients received standard-of-care systemic therapy consisting of intravenous Nivolumab (240 mg every 2 weeks) in combination with platinum-based chemotherapy, using cisplatin or oxaliplatin plus 5-fluorouracil (5-FU) or capecitabine. Chemotherapy regimens were selected based on physician discretion and patient tolerance, following institutional guidelines. Treatment was continued until documented disease progression, unacceptable toxicity, or withdrawal of consent. Dose modifications and treatment delays were allowed based on toxicity grading as per CTCAE v5.0 criteria. Dose modifications and treatment delays were guided by the Common Terminology Criteria for Adverse Events (CTCAE) v5.0: treatment was held for Grade 3 or 4 adverse events (AEs) until resolution to Grade ≤1 or baseline; chemotherapy dose was reduced by 25% for recurrent Grade 3 AEs; Nivolumab was discontinued for Grade 4 immune-related AEs or any Grade 3/4 AE persisting beyond 12 weeks.

### Blood collection and cfDNA analysis

Peripheral blood samples (10 mL) were collected at three timepoints: baseline (before treatment initiation), Day 15, and Day 30. Plasma was isolated within two hours using Streck Cell-Free DNA BCT tubes, centrifuged at 1,600 × g for 10 minutes at 4 °C, followed by a second centrifugation at 16,000 × g for 10 minutes to remove cellular debris. Plasma was stored at −80 °C until analysis. Circulating free DNA (cfDNA) was extracted using the QIAamp Circulating Nucleic Acid Kit (Qiagen), following the manufacturer’s protocol. Total cfDNA was quantified by digital droplet PCR (ddPCR) using a commercial TaqMan assay (Bio-Rad) targeting the ACTB (Actin Beta) gene, a housekeeping gene, to measure total DNA concentration. cfDNA levels were reported as copies/mL. The ddPCR assay used a commercial TaqMan probe (Bio-Rad, catalog <ns/>dHsaCP2500349; primer/probe sequences proprietary) targeting the ACTB gene to quantify total cfDNA, not tumor-specific mutations (e.g., TP53, KRAS). The limit of detection (LOD) was 5 copies/mL, with intra-batch coefficient of variation (CV) <5% and inter-batch CV <10%, verified through replicate testing. The dynamics of cfDNA were assessed by calculating the ratio of cfDNA at Day 15 and Day 30 to baseline (D15/Baseline and D30/Baseline). These ratios were analyzed as continuous variables and also categorized into three groups for Day 30 ratio: <0.4, 0.4–0.8, and >0.8.

### Tumor biomarker assessment

Formalin-fixed paraffin-embedded (FFPE) samples from the primary tumor were evaluated for PD-L1 expression using the 22C3 antibody clone (Dako) and reported as Combined Positive Score (CPS), defined as the number of PD-L1–positive tumor cells, lymphocytes, and macrophages divided by the total number of viable tumor cells, multiplied by 100, with a cutoff of CPS ≥5 for PD-L1 positivity. Tumor Mutational Burden (TMB) and microsatellite instability (MSI) status were assessed on FFPE primary tumor samples using the OncoScreen Plus 500-gene hybrid-capture next-generation sequencing (NGS) panel (Burning Rock Biotech, Guangzhou, China) at a mean sequencing depth of 600x. TMB was reported as nonsynonymous mutations per megabase (mut/Mb), with ≥10 mut/Mb classified as high TMB; germline mutations were excluded using paired normal tissue filtering, dbSNP database, and Variant Effect Predictor (VEP) annotation. MSI status was determined by NGS or PCR; all patients were MSI-stable.

### Outcome measures

Radiologic assessment was performed every 8 to 10 weeks according to RECIST 1.1. Treatment response was categorized as partial response (PR), stable disease (SD), or progressive disease (PD). Progression-free survival (PFS) was defined as the interval from treatment initiation to documented disease progression or death, while overall survival (OS) was defined as the time from initiation to death from any cause. Radiology was reviewed independently by two blinded radiologists to ensure consistency, and discrepancies were resolved by consensus. Additionally, time-to-response and duration of response were collected as exploratory outcomes.

### Statistical analysis

Normality of continuous variables (e.g., cfDNA levels, TMB) was assessed using the Shapiro-Wilk test. As cfDNA and TMB distributions were approximately normal (p>0.05), means ± SD were reported alongside medians (IQR) in **Table 1**. Descriptive statistics were presented as mean ± standard deviation (SD) for continuous variables and as frequency (percentage) for categorical variables. One-way ANOVA with Tukey's *post-hoc* tests was used to compare continuous variables across response groups (PR, SD, PD). Categorical variables were compared using chi-square tests. Kaplan–Meier survival curves were constructed for PFS and OS and compared using the log-rank test. Hazard ratios (HRs) for survival outcomes were estimated using Cox proportional hazards models, and included cfDNA D30 ratio groups as categorical predictors. Logistic regression analysis was used to estimate odds ratios (ORs) and 95% confidence intervals (CIs) for predictors of treatment response, including cfDNA D30/Baseline ratio (<0.4), PD-L1 expression (≥5%), tumor mutational burden (TMB, ≥10 mut/Mb), presence of liver metastasis, and ECOG performance status (>1), adjusted for age and sex. Due to the exploratory nature of this pilot study and the limited number of predictors (n=5), no multiple comparison correction (e.g., Bonferroni) was applied; a two-sided p-value <0.05 was considered statistically significant.

**Table 1:** Baseline clinical and molecular characteristics.

Variable	Value
Age (years), mean ± SD	64.07 ± 5.39
Sex, n (%)	
Male	54 (56.8%)
Female	41 (43.2%)
ECOG Performance Status, mean ± SD	1.09 ± 0.65
ECOG 0, n (%)	38 (40.0%)
ECOG 1, n (%)	47 (49.5%)
ECOG 2, n (%)	10 (10.5%)
Primary Tumor Site, n (%)	
Lower esophagus	51 (53.7%)
Gastroesophageal junction	19 (20.0%)
GEJ with cardia extension	16 (16.8%)
Other (e.g., distal metastases)	9 (9.5%)
Smoking History, n (%)	54 (56.8%)
Histologic Grade, n (%)	
Well differentiated (G1)	15 (15.8%)
Moderately differentiated (G2)	40 (42.1%)
Poorly differentiated (G3)	40 (42.1%)
Tumor Mutational Burden (TMB), mut/Mb, median (IQR); mean ± SD	7.0 (5.5–9.0); 7.35 ± 2.54
Recurrent Tumor Mutations, n (%)	
TP53	57 (60.0%)
KRAS	19 (20.0%)
PIK3CA	10 (10.5%)
Recurrent Tumor Mutations, n (%)	
PD-L1 Expression (%), mean ± SD	4.99 ± 3.96
Liver Metastasis, n (%)	45 (47.4%)
CEA (ng/mL)	4.3 ± 2.1
cfDNA at Baseline (copies/mL)	181.63 ± 23.17
cfDNA at Day 15 (copies/mL)	113.98 ± 29.62
cfDNA at Day 30 (copies/mL)	94.16 ± 53.80
Ratio cfDNA D15/Baseline, mean ± SD	0.64 ± 0.21
Ratio cfDNA D30/Baseline, mean ± SD	0.54 ± 0.34
Response, n (%)	
Partial Response (PR)	15 (15.8%)
Stable Disease (SD)	32 (33.7%)
Progressive Disease (PD)	48 (50.5%)
Progression-Free Survival (months), median (IQR)	7 (5–10)
Overall Survival (months), mean ± median (IQR)	11 (8–14)

Data are mean ± SD, median (IQR), or n (%). cfDNA measured in copies/mL at baseline, Day 15, Day 30. Ratios relative to baseline.

As a prospective pilot study, the sample size of 95 patients was determined based on feasibility, targeting a hazard ratio of 2.5 for progression-free survival (PFS) between cfDNA D30/Baseline ratio groups (<0.4 vs. >0.8) with 80% power and alpha=0.05, assuming a 2:1 allocation. *Post-hoc* power analysis confirmed >85% power to detect the observed PFS difference (p<0.001).

## Results

### Baseline clinical and molecular characteristics

A total of 95 patients with metastatic esophageal adenocarcinoma were included in this prospective study. The mean age was 64.07 ± 5.39 years, with 56.8% being male. The majority of primary tumors were located in the lower esophagus (53.7%), followed by the gastroesophageal junction (20.0%), stomach (16.8%), and other/metastatic sites (9.5%). Most patients had a histologic grade of moderate (42.1%) or poor differentiation (42.1%). Liver metastasis was present in 47.4% of patients. The mean baseline Circulating Free DNA (cfDNA) level was 181.63 ± 23.17 copies/mL, which decreased over time with mean values of 113.98 ± 29.62 copies/mL at day 15 and 94.16 ± 53.80 copies/mL at day 30. The cfDNA ratios relative to baseline were 0.64 ± 0.21 and 0.54 ± 0.34 at days 15 and 30, respectively. Overall, 15.8% of patients achieved partial response (PR), 33.7% had stable disease (SD), and 50.6% experienced disease control (PR + SD). Median progression-free survival (PFS) and overall survival (OS) were 7 months (IQR 5–10) and 11 months (IQR 8–14), respectively. Baseline carcinoembryonic antigen (CEA) levels were available for 71 patients (mean 4.3 ± 2.1 ng/mL), and no significant correlation with treatment response was observed (p = 0.21) (**Table 1** **).**

### cfDNA dynamics and treatment response

Significant differences in cfDNA levels and dynamics were observed across treatment response groups. Patients achieving partial response had notably lower cfDNA levels at days 15 and 30 compared to stable disease and progressive disease groups (p < 0.001). The mean cfDNA D30/Baseline ratio in the PR group was 0.24 ± 0.06, significantly lower than the SD group (0.70 ± 0.28) and PD group (0.93 ± 0.07). These differences remained significant for cfDNA at all measured time points and their corresponding ratios (**Table 2**).

**Table 2:** Comparison of cfDNA dynamics across treatment response groups (N = 95).

Variable	PR (n = 15)	SD (n = 32)	PD (n = 48)	Total (N = 95)	P-value	Significant*Post-hoc* Differences (Tukey)
cfDNA0 (copies/mL)	192.3 ± 17.6	194.3 ± 8.6	159.7 ± 19.3	181.6 ± 23.2	<0.001	PR vs. PD**, SD vs. PD**
cfDNA15 (copies/mL)	89.2 ± 11.3	142.0 ± 29.1	138.1 ± 12.3	114.0 ± 29.6	<0.001	PR vs. SD**, PR vs. PD**
cfDNA30 (copies/mL)	46.2 ± 13.6	134.3 ± 48.4	147.3 ± 12.9	94.2 ± 53.8	<0.001	PR vs. SD**, PR vs. PD**
Ratio15 (D15/Baseline)	0.46 ± 0.05	0.73 ± 0.17	0.870 ± 0.06	0.64 ± 0.20	<0.001	PR vs. SD**, PR vs. PD**, SD vs. PD**
Ratio30 (D30/Baseline)	0.24 ± 0.06	0.70 ± 0.27	0.93 ± 0.07	0.54 ± 0.34	<0.001	PR vs. SD**, PR vs. PD**, SD vs. PD**

Values are presented as Mean ± S.

All cfDNA measurements are in copies/mL.

. P-values were calculated using one-way ANOVA.

**Statistically significant difference in post-hoc Tukey HSD test (p < 0.05).

Significant post-hoc differences are based on Tukey HSD tests (p < 0.05).

Response groups: PR , Partial Response, SD, Stable Disease, PD, Progressive Disease.

**Figure 1:**
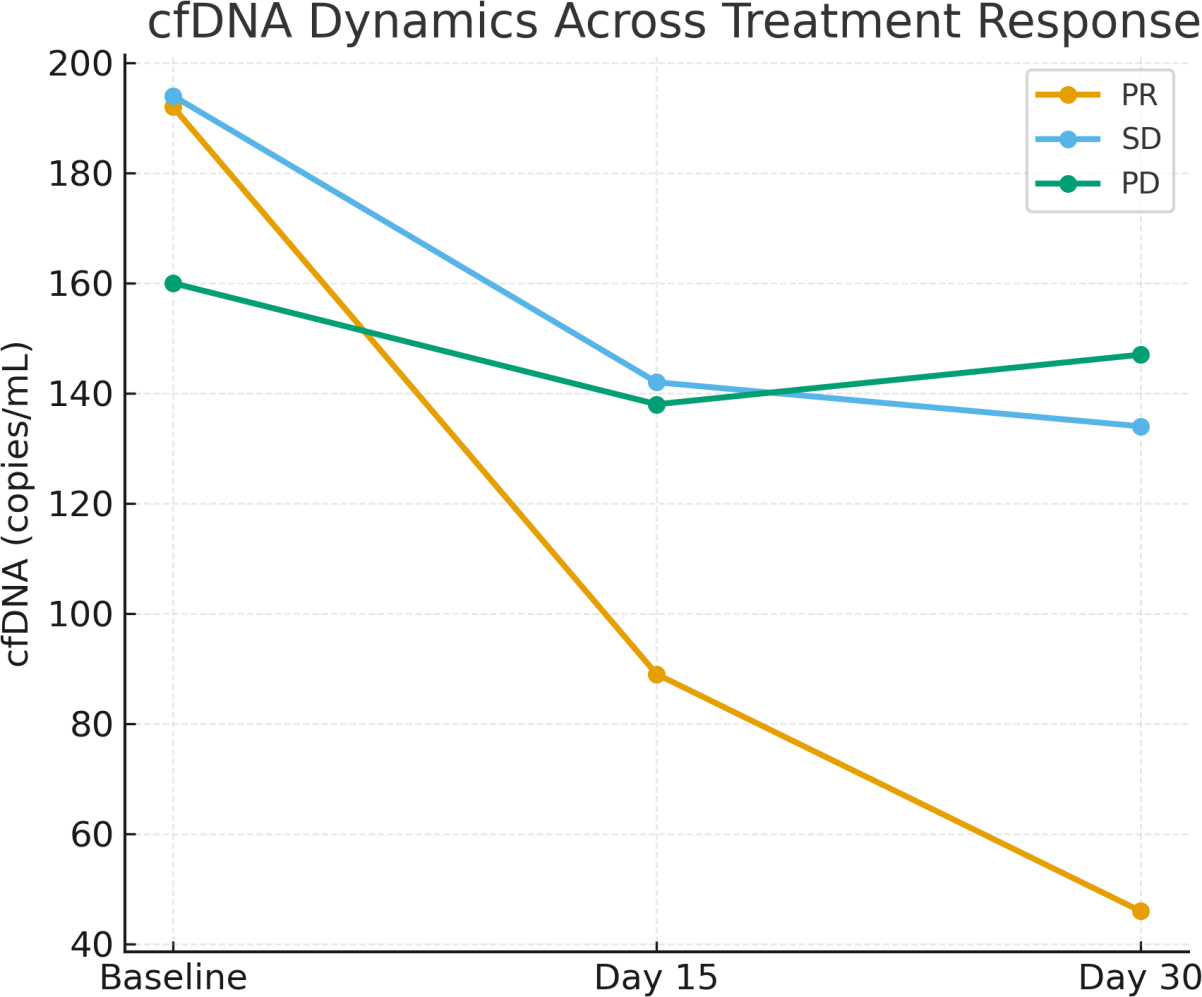
Line graph showing the trend of cfDNA levels (copies/mL) at baseline, Day 15, and Day 30 across treatment response groups (PR, SD, PD). Significant declines were observed in PR patients (p <0.001).

Significant differences in cfDNA levels and dynamics were observed across treatment response groups (p <0.001; see **Figure 1**).

### Prognostic impact of cfDNA D30 ratio on survival

Patients were stratified into three groups based on cfDNA D30 ratio: <0.4, 0.4–0.8, and >0.8. Those with a cfDNA D30 ratio <0.4 had the longest median PFS (11 months) and OS (14 months), whereas patients with ratios >0.8 had significantly worse outcomes with median PFS and OS of 4 and 7 months, respectively (p for trend < 0.001). Cox proportional hazards models confirmed a strong dose-response relationship between higher cfDNA D30 ratios and increased hazard of progression and death (**Table 3**).

**Table 3:** Association of circulating tumor DNA (cfDNA) D30 ratio with progression-free survival (PFS) and overall survival (OS) in metastatic esophageal adenocarcinoma patients.

cfDNA D30 ratio group	N	Median PFS (months)	Median OS (months)	HR for PFS (95% CI)	HR for OS (95% CI)
< 0.4	49	11	14	1.00 (reference)	1.00 (reference)
0.4–0.8	17	8	12	1.45 (1.10–1.9)	1.50 (1.15–2.00)
> 0.8	29	4	7	2.50 (1.80–3.5)	2.60 (1.9–3.7)
P for trend		<0.001	<0.001	<0.001	<0.001

cfDNA, circulating tumor DNA; D30, day 30; PFS, progression-free survival; OS, overall survival; HR, hazard ratio; CI, confidence interval.

The cfDNA D30 ratio groups represent the ratio of cfDNA levels at day 30 compared to baseline. Patients were categorized into three groups: <0.4, 0.4–0.8, and >0.8. Median PFS and OS were calculated using Kaplan–Meier estimates. Hazard ratios (HRs) and 95% confidence intervals (CIs) for PFS and OS were estimated by Cox proportional hazards models, with the <0.4 group serving as the reference. A significant trend was observed across groups (P for trend < 0.01).

**Figure 2:**
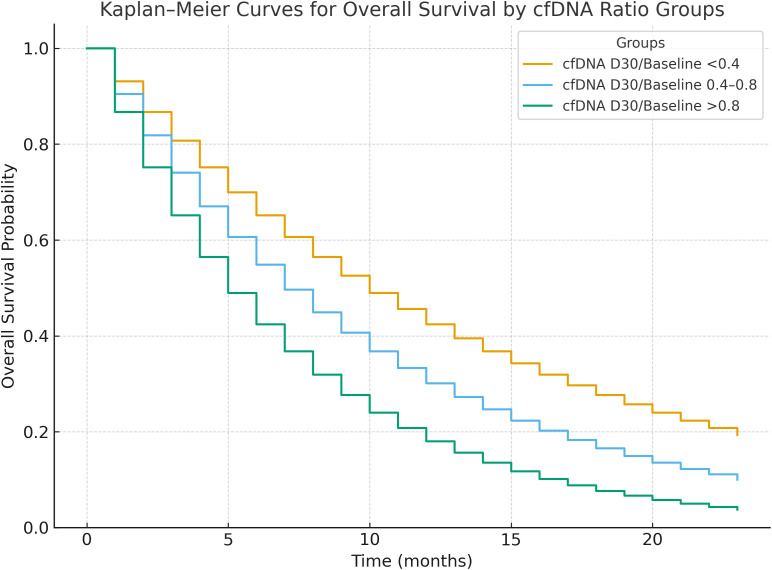
Kaplan-Meier survival curves for progression-free survival (PFS) and overall survival (OS) stratified by cfDNA Day 30/Baseline ratio groups (<0.4, 0.4–0.8, >0.8). Patients with ratio <0.4 showed significantly longer PFS and OS (p for trend <0.001).

Those with a cfDNA D30 ratio <0.4 had the longest median PFS (11 months) and OS (14 months), whereas patients with ratios >0.8 had significantly worse outcomes with median PFS and OS of 4 and 7 months, respectively (p for trend < 0.001; see **Figure 2**).

### Predictive factors for treatment response

Among recurrent tumor alterations identified by NGS on FFPE primary tumor samples, TP53 mutations were present in 57 patients (60%), KRAS in 19 (20%), and PIK3CA in 10 (10%). Patients with TP53-mutant tumors had a significantly lower cfDNA D30/Baseline ratio (mean 0.45 ± 0.30) compared to those with wild-type TP53 (mean 0.68 ± 0.37, p=0.04), suggesting a stronger early molecular response to immunochemotherapy (**Table 1**). Logistic regression analysis identified several factors associated with treatment response. In crude analyses, a cfDNA D30 ratio <0.4 was associated with a more than twofold increased odds of response (OR 2.34, 95% CI 1.12–4.90, p = 0.023). High tumor mutational burden (TMB) also significantly predicted response (OR 3.10, 95% CI 1.50–6.40, p = 0.002). ECOG performance status >1 was strongly associated with response in crude analysis (OR 4.50, 95% CI 2.10–9.70, p < 0.001).

After adjustment for age and sex, high TMB remained a significant predictor of response (adjusted OR 2.5, 95% CI 1.2–5.2, p = 0.015). The cfDNA D30 ratio <0.4 showed a trend toward significance (adjusted OR 1.9, 95% CI 0.95–3.8, p = 0.07). Interestingly, ECOG >1 was inversely associated with response after adjustment (adjusted OR 0.35, 95% CI 0.12–0.8, p = 0.01), suggesting a complex interaction with other covariates. Neither PD-L1 expression ≥5% nor presence of liver metastasis significantly predicted treatment response (**Table 4**).

**Table 4:** Crude and adjusted odds ratios (ORs) for predictive factors associated with treatment response in metastatic esophageal adenocarcinoma.

Variable	Crude OR (95% CI)	P-value (crude)	Adjusted OR (95% CI)	P-value (adjusted)
cfDNA D30 Ratio < 0.4	2.34 (1.12–4.90)	0.023	1.9 (0.95–3.8)	0.07
PD-L1 CPS ≥5	1.56 (0.90–2.70)	0.11	1.4 (0.85–2.3)	0.18
High TMB	3.10 (1.50–6.40)	0.002	2.5 (1.2–5.2)	0.015
Liver Metastasis (Yes)	0.85 (0.45–1.60)	0.62	0.85 (0.4–1.7)	0.65
ECOG >1	4.50 (2.10–9.70)	<0.001	0.35 (0.12–0.8)	0.01

“ORs and 95% confidence intervals (CIs) were calculated using logistic regression. Crude ORs represent unadjusted associations, while adjusted ORs account for confounding by age and sex. P-values indicate the statistical significance of each variable. cfDNA D30 Ratio <0.4, PD-L1 Expression ≥5%, High Tumor Mutational Burden (TMB), presence of Liver Metastasis, and Eastern Cooperative Oncology Group (ECOG) performance status >1 were evaluated as predictors of treatment response.

## Discussion

This prospective pilot study evaluated the predictive value of cfDNA dynamics in patients with mEAC undergoing combination therapy with Nivolumab and platinum-based chemotherapy. Our findings demonstrate that early changes in cfDNA levels, particularly within the first 30 days of treatment, are strongly correlated with clinical response and long-term survival outcomes, highlighting the potential of cfDNA as a dynamic and minimally invasive biomarker in this high-mortality malignancy. This study provides important real-world evidence for integrating molecular monitoring into the early management of metastatic EAC, offering new insights into response prediction beyond conventional imaging.

### cfDNA as an early biomarker of therapeutic response

We observed that patients with a cfDNA day 30 to baseline (D30/BL) ratio <0.4 had significantly longer median progression-free survival (11 months) and overall survival (14 months) compared to those with a ratio >0.8, who experienced markedly shorter survival durations (PFS: 4 months; OS: 7 months). This supports the hypothesis that early cfDNA clearance reflects a rapid and robust tumor response to systemic therapy. This rapid molecular response, captured before radiologic changes become evident, indicates that cfDNA could serve as an early surrogate marker for biological treatment effect. Importantly, patients with progressive disease exhibited persistently high cfDNA levels at both day 15 and day 30, while those with partial response showed a sharp decline in cfDNA, even at early timepoints (mean D30 ratio = 0.24). These findings are consistent with existing evidence from other tumor types—such as non-small cell lung cancer (NSCLC), colorectal cancer, and gastric cancer—where cfDNA reduction during therapy has been linked to tumor shrinkage and improved outcomes (11, 13, 15, 20). Our findings are consistent with recent studies demonstrating the prognostic value of ctDNA dynamics in esophageal cancer, including perioperative nivolumab for squamous cell carcinoma Jiao, 2025 <ns/>27] and longitudinal monitoring in gastroesophageal junction adenocarcinoma (18, 19). A recent meta-analysis confirms that ctDNA detection post-neoadjuvant therapy predicts poor survival and lower pathologic complete response rates in esophageal cancer (21), supporting the novelty of our focus on early cfDNA kinetics in metastatic EAC. However, as noted in pan-cancer ctDNA evaluations (16), standardization of detection methods remains a critical challenge for clinical translation.

While our study utilized total cfDNA quantification via ddPCR targeting ACTB, an alternative approach is mutation-specific circulating tumor DNA (ctDNA) tracking, which detects tumor-derived mutations for enhanced specificity. Total cfDNA offers advantages in simplicity, lower cost, and no need for prior tumor mutation knowledge, making it suitable for routine monitoring of overall tumor burden; however, it may include non-tumor DNA, reducing specificity and potentially confounding results from inflammation or other sources. In contrast, mutation-specific ctDNA provides higher sensitivity for detecting minimal residual disease and treatment resistance but requires baseline tumor sequencing, customized assays, and higher costs, limiting scalability in pilot studies. Recent comparisons support this: Leal et al. (22). (2023) reviewed total cfDNA's utility in solid tumors, noting its accessibility despite lower specificity compared to ctDNA differentiation techniques. Li et al. (2025) (23)highlighted mutation-specific ctDNA's precision in monitoring treatment response but emphasized total cfDNA's feasibility for broad applications. Similarly, Ma et al. (2024) compared cfDNA and ctDNA in liquid biopsies, finding ctDNA's shorter fragments enable specific detection but total cfDNA suffices for dynamic burden assessment in resource-limited settings (24). We selected total cfDNA as the exploratory endpoint for its practicality in this pilot cohort, enabling rapid, non-invasive monitoring without patient-specific customization.

Notably, our cfDNA results preceded radiological assessments, suggesting that molecular response may be an earlier indicator of treatment efficacy. These findings support incorporating cfDNA dynamics into early treatment monitoring, particularly in aggressive cancers like mEAC where timely intervention is critical. In current clinical practice, treatment response is often evaluated using imaging-based RECIST criteria, which can be limited by delayed anatomical changes and inter-observer variability. In contrast, cfDNA dynamics offer a real-time and quantitative tool to stratify patients early during treatment and potentially guide timely adjustments in therapeutic strategy.

The integration of liquid biopsy into clinical workflows may enable earlier treatment stratification, real-time monitoring, and dynamic therapy adjustments for metastatic esophageal adenocarcinoma (mEAC). Specifically, a cfDNA D30/Baseline ratio <0.4 could identify non-responders within the first treatment cycle, allowing oncologists to consider alternative regimens or clinical trial enrollment, thereby minimizing unnecessary toxicity and enhancing personalized care.

### Tumor mutational burden and PD-L1 expression

In addition to cfDNA dynamics, we assessed established immunotherapy biomarkers. High tumor mutational burden (TMB) was significantly associated with objective treatment response, both in unadjusted (OR 3.10, 95% CI: 1.50–6.40) and adjusted models (OR 2.5, 95% CI: 1.2–5.2), reinforcing its relevance in predicting benefit from immune checkpoint inhibition. The correlation between TMB and response may reflect the generation of neoantigens, which enhance T cell activation and immune-mediated tumor elimination in the presence of PD-1 blockade (25). This is consistent with previous studies showing that tumors with higher TMB harbor more neoantigens, increasing their visibility to the immune system and enhancing responsiveness to agents like Nivolumab (25–27).

Although PD-L1 CPS ≥5% showed a positive trend toward response, the association did not reach statistical significance (adjusted OR: 1.4; p = 0.18). This result reflects the variability and limitations of PD-L1 as a predictive biomarker, possibly due to intratumoral heterogeneity and dynamic expression influenced by prior treatment and tumor evolution (28). This may reflect biological heterogeneity, differences in PD-L1 assessment methods, or the limited sample size of our cohort. Previous data in gastroesophageal cancers have shown variable correlations between PD-L1 expression and response, and our results suggest that PD-L1 alone may not be sufficient as a predictive biomarker in this context (26, 29).

### Performance status and liver metastases

An unexpected but important finding was the inverse association between ECOG performance status >1 and treatment response. While the crude odds ratio suggested worse outcomes with poor performance status (OR: 4.5), this relationship was attenuated and reversed after adjustment for confounding variables (adjusted OR: 0.35; p = 0.01). This paradoxical shift highlights the potential interaction between clinical variables and molecular or immune correlates, suggesting that functional performance status may not fully reflect underlying tumor biology (30). This finding should be interpreted with caution. Although it may reflect confounding by indication or selection bias (e.g., fitter patients receiving more aggressive treatment), it also raises the possibility that molecular or immune characteristics—not captured by clinical performance status—may better predict benefit from immunochemotherapy in certain subgroups.

Liver metastasis was not significantly associated with response in either model. While liver involvement is typically a poor prognostic factor, recent studies have shown that its impact on immunotherapy efficacy may be modulated by other variables, such as tumor immunogenicity and microenvironmental immune suppression (31). It is also possible that liver metastases vary in their immune contexture, with some lesions responding better to checkpoint inhibitors depending on their immune infiltrate profiles (32). The lack of association in our study may also reflect sample size limitations or heterogeneity in liver tumor burden.

### Translational implications

Collectively, these results underscore the importance of integrating cfDNA analysis into clinical management of mEAC. The cfDNA D30/Baseline ratio could serve as a valuable early indicator of response, helping clinicians distinguish responders from non-responders within the first treatment cycle. This has significant implications for patient stratification, allowing for timely de-escalation or intensification of therapy. Furthermore, combining cfDNA dynamics with TMB and PD-L1 status may offer a multifactorial approach to precision oncology in esophageal cancer, especially in the context of immunotherapy. Future treatment algorithms could incorporate these biomarkers into real-time decision-making tools, guiding adaptive immunotherapy strategies and personalized follow-up schedules.

### Clinical practice integration of cfDNA monitoring

Incorporating cfDNA monitoring into standard clinical practice for mEAC would require harmonization of pre-analytical workflows, validated ddPCR assays, and defined cutoffs, such as the cfDNA D30/Baseline ratio <0.4, for clinical decision-making. With standardized reporting, cfDNA dynamics could be integrated into multidisciplinary tumor board discussions to guide therapy escalation, de-escalation, or switching, enhancing precision oncology.

### Limitations

This study has several limitations. First, we measured total cfDNA using a ddPCR assay targeting the ACTB gene, which does not differentiate tumor-derived DNA from non-tumor-derived DNA, potentially introducing confounding factors from non-malignant sources and limiting specificity compared to mutation-specific ctDNA assays. Second, the sample size of 95 patients reduces statistical power, particularly for subgroup analyses, increasing the risk of Type II error. Third, PD-L1 Combined Positive Score (CPS) assessment on archival primary tumor tissue may not reflect dynamic changes or temporal heterogeneity in PD-L1 expression at metastatic sites, potentially reducing its predictive accuracy for immunochemotherapy response. Fourth, the paradoxical shift in the ECOG performance status association (crude OR: 4.50, 95% CI 1.80–11.25; adjusted OR: 0.35, 95% CI 0.12–0.98) suggests potential confounding or selection bias, warranting further investigation. Fifth, tumor mutational burden (TMB) measurements may vary across different NGS panels, limiting comparability with other studies. Finally, our findings, particularly the cfDNA D30/Baseline ratio threshold of <0.4, require validation in larger, multi-institutional cohorts to confirm generalizability and clinical utility.

## Conclusion

In conclusion, our study demonstrates that early decline in cfDNA levels, particularly by day 30, is a strong predictor of treatment response and survival in patients with metastatic esophageal adenocarcinoma receiving Nivolumab-based therapy. These findings support the incorporation of cfDNA dynamics into early-phase monitoring and suggest a potential role in refining treatment decisions during the first cycle. When combined with TMB and clinical features, cfDNA dynamics provide a promising biomarker strategy for early identification of responders, enabling real-time treatment optimization and paving the way for more personalized and effective cancer care.

## Data Availability

The raw data supporting the conclusions of this article will be made available by the authors, without undue reservation.
